# Hemoptysis from Rasmussen’s Aneurysm

**DOI:** 10.4269/ajtmh.21-1306

**Published:** 2022-05-02

**Authors:** Akira Kawashima, Tomoki Hirose, Masahide Horiba

**Affiliations:** Department of Respiratory Medicine, National Hospital Organization Higashisaitama National Hospital, 4147, Kurohama, Hasuda, Saitama, Japan

**Figure 1.**
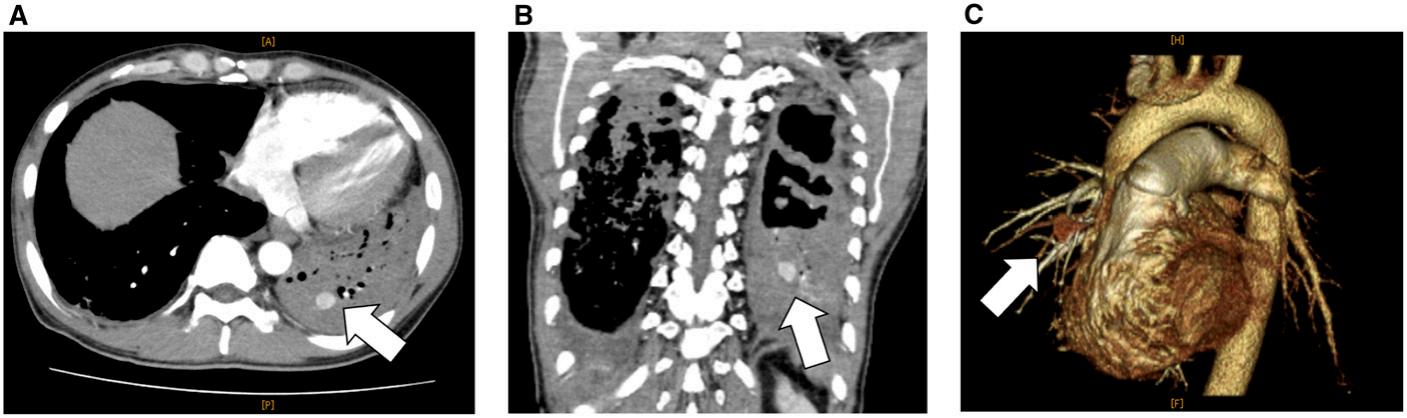
Axial view (**A**) and coronal view (**B**) of contrast-enhanced computed tomography (CT) and CT angiography with three-dimensional reconstruction (**C**) showing an 11-mm-diameter pseudoaneurysm in the left lower pulmonary artery (white arrow) surrounded by high-density fluid collection. This figure appears in color at www.ajtmh.org.

**Figure 2.**
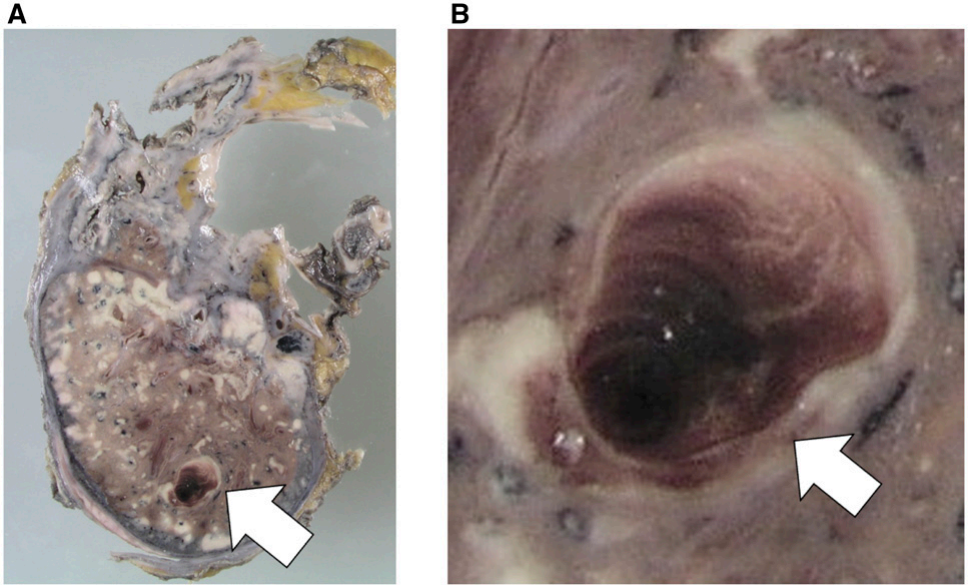
(A) Histopathology of the necrotic layer of the left lung showing hemorrhage (white arrow); (**B**) close-up view (white arrow). This figure appears in color at www.ajtmh.org.

**Figure 3.**
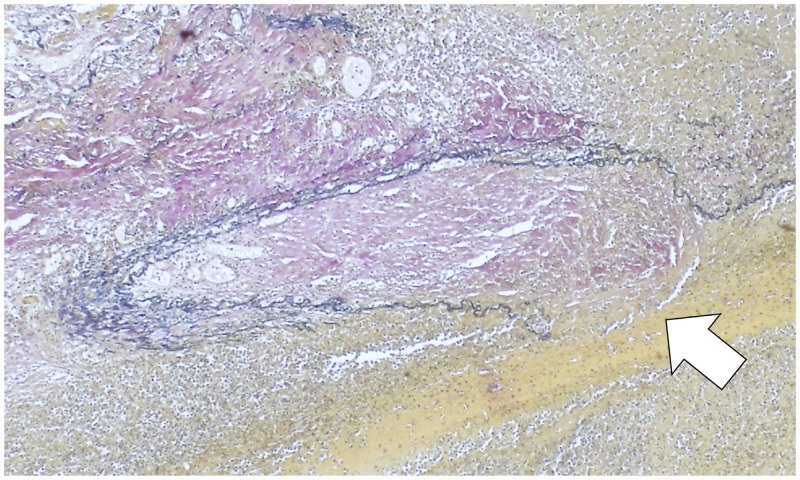
Elastic van Gieson–stained lung specimen from autopsy showing elastic fibers that were destroyed by inflammation and became fragile; the white arrow indicates rupture. The collagen fibers are organized, and the blood vessels are recanalized. A few multinucleated giant cells are present outside the elastic fibers (magnification, ×100). This figure appears in color at www.ajtmh.org.

A 43-year-old Japanese man with newly diagnosed type 2 diabetes mellitus was admitted to our hospital for the management of pulmonary tuberculosis; he had sputum production, cough, and exertional dyspnea for 3 months. The sputum culture and polymerase chain reaction test results were positive for *Mycobacterium tuberculosis*. The patient was commenced on directly observed antituberculosis therapy, including isoniazid, rifampin, ethambutol, and pyrazinamide. Forty-five days after treatment commenced, he had hemoptysis of approximately 100 mL, which was conservatively managed with hemostatic agents, including tranexamic acid and carbazochrome sodium sulfonate hydrate. Contrast-enhanced computed tomography (CT) and CT angiography (Figures 1A–C, white arrow) with three-dimensional reconstruction were performed on the same day, and the findings showed an 11-mm-diameter pseudoaneurysm of the left lower pulmonary artery surrounded by high-density fluid collection. On the 60th day of hospitalization, another hemoptysis episode occurred, and his respiratory condition deteriorated rapidly. Three days later, his blood oxygen saturation gradually dropped, and he received oxygen via a reservoir mask. Three hours later, he developed ventricular fibrillation, and despite intensive care with endotracheal intubation, replacement fluids, defibrillation, and noradrenaline, his heart rate did not normalize, and he died of cardiac arrest. Autopsy revealed hemorrhage within a necrotic layer in the left lung (Figure 2A and B, white arrow). Elastic van Gieson staining showed fragile elastic fibers destroyed by inflammation (Figure 3, white arrow). This rupture of the pulmonary artery pseudoaneurysm caused by *M. tuberculosis* infection is called Rasmussen’s aneurysm.

Rasmussen’s aneurysm was first described in 1868 in 11 patients with tuberculosis^1^; since then, more than 200 cases have been documented in the literature to our knowledge. On the basis of autopsies, the prevalence of Rasmussen’s aneurysm has been estimated to be 4% in patients with pulmonary tuberculosis.^2^ In a study of massive hemoptysis associated with tuberculosis infection diagnosed using CT, Rasmussen’s aneurysm was found in 6.9% of patients.^3^ There have been reports of mortality due to massive hemoptysis, as observed in the present case.^4^ Apart from the resection of the affected pulmonary lobe, endovascular embolization has emerged as the most successful and less invasive treatment option in recent years.^5^ Our hospital did not have an interventional radiologist. In addition, the patient’s respiratory condition was poor, making patient transfer to another hospital difficult. If contrast-enhanced CT and CT angiography had been performed earlier, Rasmussen’s aneurysm could have been diagnosed earlier, and the patient’s life could have been saved by performing endovascular embolization.
